# Why hand hygiene is not sufficient: modeling hygiene competence of clinical staff as a basis for its development and assessment

**DOI:** 10.3205/zma001247

**Published:** 2019-08-15

**Authors:** Martin Gartmeier, Maria Baumgartner, Rainer Burgkart, Susanne Heiniger, Pascal O. Berberat

**Affiliations:** 1 Technical University of Munich, School of Medicine, Klinikum rechts der Isar, TUM Medical Education Center, Munich, Germany; 2 Technical University of Munich, Klinikum rechts der Isar, Department of Orthopedics and Sports Orthopedics, Munich, Germany

**Keywords:** hygiene, hand hygiene, hygiene competence, situational judgment test, simulation

## Abstract

Adhering to hygiene standards in daily clinical work is an important characteristic of qualitatively high-value medical care. In this regards, hand hygiene is often focused on in the literature. From the viewpoint of medical education research, we argue that this focus is too narrow to explain how staff who are working clinically with patients implement and adhere to standards of hygiene across a wide variety of tasks of their daily clinical routine. We present basic features of a differentiated concept of *hygiene competence*, which includes specialized knowledge, corresponding inner attitudes, and action routines that are customized to the needs of specific situations. Building on that, we present a current simulation-based course concept aimed at developing hygiene competence in medical education. Furthermore, we describe a test instrument that is designed according to the principle of a* situational judgment test* and that appears promising for the assessment of hygiene competence. The course and the measurement instrument are discussed in regards to their fit to the competence model and the related perspectives for research and teaching.

## Zusammenfassung

Die Einhaltung von Hygienestandards im klinischen Arbeitsalltag ist ein wichtiges Merkmal qualitativ hochwertiger medizinischer Versorgung. Diesbezüglich wird in der Literatur häufig der Aspekt der *Handhygiene* fokussiert. Aus Sicht der medizinischen Ausbildungsforschung argumentieren wir, dass dieser Fokus zu kurz greift, um zu erklären, wie klinisch an Patientinnen und Patienten tätige Personen Hygienestandards über verschiedenste Tätigkeiten ihres klinischen Alltags hinweg einhalten und umsetzen. Daher entwickeln wir Grundzüge eines differenzierteren Konzepts von *Hygienekompetenz*, das spezialisiertes Wissen, entsprechende innere Einstellungen sowie auf die Anforderungen spezifischer Situationen zugeschnittene Handlungsroutinen umfasst. Darauf aufbauend stellen wir ein aktuelles, simulationsbasiertes Kurskonzept zur Förderung von Hygienekompetenz in der medizinischen Ausbildung vor. Außerdem beschreiben wir den Aufbau eines Testinstruments, das nach dem Prinzip eines *Situational Judgement Tests* gestaltet und für die Erfassung von Hygienekompetenz vielversprechend ist. Kurs und Messinstrument werden hinsichtlich ihrer Passung zum Kompetenzmodell sowie den damit verbundenen Perspektiven für Forschung und Lehre diskutiert.

## Introduction and problem statement

Hygiene in daily clinical work hardly is a new topic, but nonetheless is currently much debated in a controversial way, mainly regarding the prevention of infections in the treatment of patients [1]. Various aspects play a role here, either legally or economically [2]. Foremost, hygiene is directly related to the maxim, “first, do no harm” [3] as the fundamental obligation to organize medical and nursing care in such a way that no harm for patients results from it. More precisely, one could formulate two equally important goals of medical intervention: first, the successful treatment of patients for the relief of their suffering; second, the avoidance of possible suffering and troubles that arise *from the medical treatment itself* under all circumstances. In reality, these two aspects often cannot be separated from each other. Therefore, hygiene should be considered and incorporated as an integral part of any medical practice. For example, if a needle is placed hygienically correct under absolutely sterile precautionary measures, but perforates the lungs instead of the subclavian vein, a pneumothorax can be the serious consequence. At the same time, despite a correct puncture of the vein, a hygienically mistaken (unsterile) procedure can lead to harm for the patient, e.g. through infection.

In view of the importance of the ability to carry out clinical activities under strict observance of hygiene guidelines, it is worrisome that the topic of hospital hygiene is barely focused in medical education research [4]. The competence of health care staff working with patients in hospitals, private practices, etc. is the most important prevention factor against the spread of infections and the most important resource for the adherence to hygiene guidelines. The existence of a number of studies on the effectiveness of training programs for hand hygiene show that in principle, consciousness for this situation exists [5], [6], [7], [8], [9]. Also, various instruments measuring hygiene-related behaviors and attitudes have been developed [10], [11], [12]. However, the demand has been expressed for more intensively educating and training healthcare personnel in the area of hygiene [4], [13].

We argue however that the focus on hand hygiene is too narrow, in view of the complexity and the demands of clinical practice. People working clinically carry out a multitude of tasks in which they encounter very specific demands in regards to the adherence to hygiene standards. Thus, even during seemingly simple everyday duties (such as taking a pulse or drawing blood from non-infectious patients), there is a multitude of possibilities to transmit germs, many of these possibilities have no direct relationship to the topic of hand hygiene. For instance, the topic of clothing plays a role: a doctor’s smock coming in contact with a hospital bed is a proven possibility to transport germs from one patient to the next [14]. Furthermore, neckties, watches, jewelry, and artificial fingernails contain hygiene risks [15] which are related to the topic of hand disinfection but also go beyond it. Also there are many hygiene guidelines for putting on sterile OR clothing [16] that go beyond basic hand disinfection, e.g. in regards to hand position while dressing (hands above the belt line), the movement radius of sterilely dressed persons, or the contact of these persons with objects located in the OR area (e.g. the instrument table). In the daily clinical routine, there are many specific situations and work processes during which hygiene must be maintained over sequences of complex steps of work. In such situations, transmission of germs can occur, even despite carefully performed hand disinfection before and after the procedure. So, despite basic hand disinfection is performed regularly and properly, a multitude of possibilities exist in the context of the daily clinical duties to breech hygiene rules, spread germs, and endanger patients (and medical personnel).

From this perspective, we argue that it is not sufficient if clinical staff (medical, nursing, and therapeutic) perform hand hygiene. We view hand hygiene as an important part of a more sophisticated competence that includes various aspects. Here is a gap in the medical education research. So far, no sound concept exist in this field that describes which personal resources clinical staff actually use in order to work hygienically. A glance at the German National Competence-Based Learning-Goal Catalog of Medicine (NKLM, [http://www.nklm.de]) confirms this notion. This document mentions hygienic hand disinfection as a learning goal (14b.1.1.1). Beyond that though, it describes specific areas (e.g. the OR, 14b.1.13) and activities (such as changing bandages, 14b.1.1.4), for which graduates should develop specific abilities for adherence to the standards of hygiene. Thus also in the NKLM, an area of physician competence critical to hygiene is addressed that goes beyond thorough and regular hand disinfection.

So far, there is only sparse knowledge about promising strategies for the promotion and assessment of this competence. Both aspects are, in our opinion, equally important and closely related to each other. In order to be able to reliably evaluate the success of training concepts, corresponding measurement instruments are indispensable: “the measurement of competence has (…) a key function in the optimization of teaching processes and for the further development of educational systems” [17]. As the second half of this citation indicates, instruments for the measurement of competence can be used, in order to assess the effectiveness of didactic measures, but also in order to carry out broader measurements of competence and, building on that, to identify needs for training and continuing professional development. On this basis, we argue that a theoretical model is helpful and necessary that captures the complex and multidimensional nature of the ability to work hygienically in clinical practice [17], [18].

The first focus of this contribution therefore is the description of such a competence model. It describes the personal resources that serve as basis of the ability to complete complex clinical processes in compliance to hygiene standards. For this purpose, we use the term “hygiene competence”, which has already been occasionally used in the pertinent literature [19], [20], [21]), but so far without a differentiated, theoretically grounded underlying conception. In order to close this gap, we use a pedagogical-psychological understanding of *competence as personal disposition* [22] as our starting point (see the following section). In order to make the added value of the model clear, its application will be shown in two concrete perspectives: on the one hand regarding the *promotion* of this competence through simulation-based training concepts, and on the other hand, regarding the *assessment* of this competence through a situational judgment test (SJT).

## Hygiene as a professional competence of clinical staff

First, we discuss the conception of the term competence underlying our model. We understand competence as “realized abilities” [http://www.nklm.de] [22], [23] – i.e., personal dispositions that are applied in situations which are typical for a specific professional field. These two characteristics of competence are best described drawing upon the concepts of *multidimensionality* (of the personal dispositions) and *context-dependency* (of the situations).

Context-dependency describes the assumption that for a work context, typical situations or work-tasks exist for which specific demands can be described. In regards to hygiene, that means describing situations typical for a particular work context in which hygiene plays an important role and for which specific requirements exist. If one thinks for example of surgeons, they must be able to master various situations inside the OR (e.g. dressing for the OR, working in the OR, leaving the OR) and outside the OR (e.g. taking blood from patients, physical examination of patients) in hygienically correct ways. Indeed, the general guidelines on hospital hygiene apply for all these situations. However, each situation poses (more or less complex) specific demands. For instance, a particular procedure exists for entering the OR in a hygienically correct way and behavioral routines can be precisely specified for that situation in order to ensure that hygiene standards are adhered to. A person who is competent in regards to hygiene is therefore capable of successfully completing the typical situations of his or her specific area of work in an hygienically correct way.

We argue that a person who is competent in this respect has various personal resources, i.e. characteristics and dispositions, which are the basis for successful performance in professional situations. As already mentioned, these personal resources are *multidimensional*. That means they encompass various aspects: typically, these aspects are specialized knowledge, specific attitudes, and practical skills [12]. For example, in order to uphold the strict hygiene guidelines in the OR, it is necessary to be familiar with the pertinent hygiene rules. Furthermore, background knowledge from the subjects of microbiology or virology is important. Yet knowledge alone is not sufficient. The person must also be familiar with the corresponding processes of action and be capable of carrying these out correctly. Moreover, the person should have certain inner attitudes corresponding to the importance and significance of hygiene, in order to maintain the necessary diligence even in situations of high workload and of many competing demands [24]). Ideally, these aspects merge in clinical professional behavior which is in correspondence to hygiene guidelines (see table 1 (Tab. 1)).

The three facets of competence sketched in the model – knowledge, attitudes, and behavioral routines – will be described in more detail in the following.

### 1. Knowledge

Today detailed subject knowledge from various disciplines exists, e.g. microbiology and infectiology, that is relevant to clinical hygiene [25]. Furthermore, in regards to the concrete application of this knowledge in clinical contexts, knowledge about legal questions regarding hospital hygiene, recommendations for how to prevent hospital-acquired infections, and concrete plans and guidelines to ensure hospital hygiene is relevant [2]. Moreover, there is a great number of clinical studies [26], [27], [28] and reviews [9], [18] on various aspects of hygiene that contain information relevant for clinicians. Well-grounded knowledge of this multifaceted literature represents a foundation for hygiene competence. Also, explaining under which conditions and in which ways certain germs multiply or spread as well as which pathological consequences could result, is helpful and important regarding hospital hygiene. Regarding the demand to hygienically carry out practical activities in the clinical context, however, complementary practical behavioral knowledge is necessary (e.g. placing a urinary catheter, changing bandages, handling of infectious patients, etc.).

#### 2. Attitudes

Various empirical studies have shown that specific inner attitudes are co-determining the degree to which subject knowledge about hygiene is applied and the corresponding ways of acting are actually practiced in the clinical routine [18]. A recent empirical study [29] has shown that physicians practice hand hygiene in their daily work more frequently after a one-hour mindfulness-intervention. The focus of this intervention was neither specialized subject knowledge nor particular abilities, but more consciousness and presence in the daily work. Another study [30] has shown that awareness of being observed regarding hand hygiene, being a role model for others, and positive attitudes towards hand hygiene are connected with more frequent and more thorough hand disinfection.

Carrying out clinical activities under careful adherence to hygiene standards is cumbersome in many situations, costs time, and slows down the pace of work. A workday that is characterized by a multitude of competing demands certainly contributes to a reduction of the amount and carefulness of hygiene measures practiced by clinical personnel [28], [31]. So it is necessary to maintain attitudes despite existing pressures of time and organization and to consequently practice hygiene measures. 

Regarding attitudes about hygiene, it has been shown that self-reported attitudes and behaviors often do not correspond with the behaviors actually observed [12]. Accordingly, professional routine seems to sometimes influence attitudes and ways of acting so that people working clinically actually carry out fewer hygiene measures than they believe they do or report doing in a survey [26], [32]. Therefore, the question is relevant how attitudes towards hygiene as one aspect of hygiene competence can be assessed. A relevant questionnaire that has already been widely validated [12] covers the following aspects:

1. a proactive attitude toward the effectiveness of hygiene,
2. belief in one’s own abilities to behave in a hygienically correct manner (expectation of self-efficacy),
3. imitation of hygiene-related ways of behaving of other persons,
4. self-regulation of one’s own hygiene behavior, and (e) effectiveness of reinforcing and inhibiting factors.

#### 3. Skills and ways of acting

Persons who are working clinically on and around patients are able to complete their work successfully in *a hygienic manner* through specific practical skills and ways of acting. That means that they protect patients, other people in their vicinity (colleagues, family members), and themselves from the transmission of germs, pathogens, and infections. This includes rather general ways of acting, such as e.g. the regular disinfection of their hands according to the current guidelines [33], [34], [35], taking off jewelry, and not wearing artificial fingernails at work. Furthermore, there are medical/nursing activities for which those kinds of general hygiene measures are equally necessary, but which are not sufficient to carry out those activities hygienically. For example, to place or remove a urinary catheter, *situation-specific ways of acting* are necessary, which must each time be carried out correctly and in the right sequence. 

On the basis of this first draft of a model of hygiene competence, various follow-up questions can be formulated. How can the model be specified further? How can hygiene competence be promoted through targeted didactic interventions? How can measurement instruments be designed that are suitable for *assessing* this competence? These questions permit very different answers and offer many opportunities for further research. However, the following sections will show how the sketched concept of hygiene competence can be developed further (e.g. through consideration of contextual factors) and what a simulation-based promotion of hygiene competence could look like practically. Furthermore, we describe how an assessment of this competence with the aid of a situational judgment test is possible.

## Further development of the model of hygiene competence and consideration of contextual factors

In order to substantiate the proposed model of hygiene competence, personal resources should be identified on the basis of which clinical staff implement hygiene in daily work. Here, various starting points are promising. First, various recent studies pursue the question which knowledge or attitudes clinical staff refer to when they describe their own behavior on the topic of hygiene in the clinical daily routine [24], [36], [37], [38]. A deeper, systematic reprocessing of this literature can provide an insight into central components related to this. Second, an empirical approach is also promising, e.g. through questioning experts for hospital hygiene or through observation of medical/nursing staff in the workplace [11], [26], [30]. Third, the theoretical relation to current debates on competence is not yet completely worked out in the present text. A deeper use of these approaches, as well as of other psychological explanatory models [32], thus seems to be promising, in order to develop the basis for the synthesis of existing evidence as well as for new research results [39].

Fundamentally, observing the clinical behavior of individuals outside of the context in which it occurs makes little sense. The contextual conditions within a hospital or a practice influence the degree to which standards of hygiene are adhered to. Thus, in addition to the model of hygiene competence, we propose a contextual model that classifies various influence factors according to whether they hinder, permit, or promote hygienic clinical behavior (cf. table 2 (Tab. 2)).

The factors described in the model as *enablers* represent necessary but not sufficient conditions. They include essential structural framework conditions through which competence resources of clinical personnel can be realized. They include personnel structures (sufficient number and competence of personnel, [24]), material and spatial conditions (sufficient number of disinfectant dispensers, reasonably placed sinks, hospital clothing, [14], [24], [40], [41]), and guidelines on the topics of clothing, jewelry, and fingernails (artificial nails, nail grooming, [14], [40], [41]). First and foremost, the provision of these enablers lies in the responsibility of the institution.

Furthermore, the model includes *promoters*, in the sense of both necessary and sufficient factors for the adherence of hygiene standards. These factors contribute to implementing hygienic behavior in the clinical daily routine, on the basis of existing framework conditions (i.e. *enabler* factors). This includes factors that contribute to the promotion of hygiene competence, e.g. workshops or training sessions [9], [28], [41]). Besides, communication seminars for employees can also be beneficial – especially when they aim at maintaining hygiene rules within the team (nursing staff, therapists, physicians, [42]). Furthermore, increased attention promotes hygiene competence, for example implemented through evaluations, mentoring, feedback, institution-internal and national/international campaigns [1], [41]) or awareness-focused programs [29]. A further aspect important in this respect are role-models, who influence young clinicians in their behavior [30], [31], [43], [44]. It is very important that these models work hygienically correct [31], [36]. In order to implement promoters, instruments can be used to detect the most important reasons for non-adherence to hand hygiene and for custom-fit implementation of corresponding interventions [45].

Finally, we propose to include *barrier* factors in the contextual model, which impede an adequate adherence to hygiene guidelines. Such barriers include for example a very high workload, too few personnel, or high time pressure [24], [30], [33]. Also material and spatial structures, such as for example a lack of disinfection fixtures or impractically placed sinks, can be obstructing influence factors [24], [40]. Activities that carry a high risk of cross-transmission (germ spreading), as well as specific technical-medical procedures (in the OR, anesthesia theater, emergency room, or the ICU) seem to be risk factors for non-adherence of hygiene measures [31]. Negative role-models can be a further barrier [31], [43]. Since positive role-models seem to be rather lacking currently, role-models are mentioned both among the *promoters*, when present, and among the *barriers*, when not. In order to achieve behavioral change among clinicians, it is recommended to actively identify barriers (e.g. through questionnaires, interviews with personnel, etc.) and to initiate change processes on that basis [9]. The influence factors mentioned (enablers, promoters, and barriers) should be assessed along with hygiene competence to investigate relationships with this competence. 

## Promotion of hygiene competence – simulation course at the TUM Medical Education Center

Basic hygiene competence should be established already during undergraduate medical education. In this respect, the multifactorial competence model presented here suggests that various aspects should be focused, especially basic knowledge, practical behavioral knowledge and relations to relevant clinical situations. In the literature, various didactic approaches are currently described for the acquisition and improvement of hand hygiene [5], [6], [46], [47]. Approaches that combine various training methods with each other seem to be more effective, also in the long-term [1], [6], [9]. Therefore we argue that simulation-based approaches – also in non-virtual rooms – are for various reasons very promising for the promotion of hygiene competence. Complex clinical processes can be re-enacted that present high demands in regards to hygiene and enable a fusion of knowledge and action in the way described previously. Simulations take place in protected spaces, in which mistakes remain without negative consequences and which permit reflection on the situation on the basis of feedback and discussion.

In the following, a simulation-based hygiene course^1^ will be described, which was developed at the Rechts der Isar Hospital of the Technical University of Munich and which is part of the local medical curriculum. In this course, students learn to work hygienically correct in specific clinical scenarios and to assess potential sources of infection. The course consists of a basic and an advanced module, each comprises four clinical scenarios with the themes listed in table 3 (Tab. 3).

As an example, we describe scenario 1, “urinary catheter” from the basic course:

*The students treat a male patient with urinary retention. In order to enable the removal of urine, the sterile placement of a catheter is practiced on a catheterization model. The required utensils are present in the room. At the beginning of the scenario, general aspects are discussed (e.g. prostate hyperplasia, latex allergies, etc.) The removal of urine then takes place by draining the urine into a container. The students must insert the catheter into the urethra in a sterile way and also avoid contamination of surroundings. To achieve this task, the students should work together in teams and actively include their assistants. Many aspects of what is learned are also transferable to other invasive procedures*.

Each course includes 24 students, who rotate through the four scenarios in small groups of 6 persons each. For each scenario, 45 minutes are available (introduction: 5 min, practice: 10 min, debriefing: 25 min, scenario rotation: 5 min). The participating students’ roles are defined in the following way: two students take the role of actors and carry out the provided assignment as correctly as possible, whereby one person takes the active role and the other person assists. Two *observers* watch and assess the activity of the actors. The observation should focus on mistakes in the performance, but should also contain alternative possible solutions and confirmation of correctly performed actions. Two further students act as *superminds*. Their job is to formulate an ideal solution for the provided assignment. Thereby, they do not relate directly to the performance in the simulation, but present their solution independently. Across the various scenarios, the students switch roles, so that every student adopts each role at least once. What is common to all roles is that they do not have access to guidance or model solutions, so they must themselves figure out hygienically correct ways of proceeding. During and at the end of the various scenarios, a short group discussion with feedback takes place each time. The course satisfies a learning principle successfully established in the context of hygiene interventions, i.e., to combine behaviorally oriented feedback [46], [47] with different teaching approaches [6].

Both hygiene courses have received good to very good evaluations by students. In summary of a total of 461 evaluations of course 1 (basic module) that have been returned over four semesters (summer 2017, winter 2017/2018, summer 2018, winter 2018/2019), the mean grade is 1.4 (*SD*=0.24, with a school grading scale of 1-6 and 1 being the best grade). For course 2 (advanced module), we were able to analyze 350 student evaluations from the same semesters, the mean grade was 1.9 (*SD*=0.50).

In regards to the competence model described earlier, the course aims at two of the facets depicted (cf. table 1 (Tab. 1)). On the one hand, concrete behavioral routines are developed through which specific clinical activities and situations can be completed hygienically. On the other hand, the instructors provide information about clinical hygiene standards and relevant scientific evidence relevant for these situations. The third competence facet, attitudes toward hygiene, is addressed indirectly. The fact that an obligatory course in the medical curriculum is dedicated to the topic fosters the perception of hygiene as a very relevant aspect of medical professionalism. Furthermore, attitudes toward hygiene play a role in the group discussions, in which also aspects such as temporal and organizational contextual conditions and impediments in actual professional daily routine are discussed. In this way, all three competence facets are addressed within the course, and their synthesis in concrete clinical actions can be focused.

It is an open question how effective the course is regarding the development of the participants’ hygiene competence. A closely related question is how well the students manage to apply this competence beyond the course, in their daily clinical routine. The main challenge in regards to answering this question is the development of an instrument that enables a reliable and standardized assessment of hygiene competence. Such an instrument is currently being developed, the measurement concept will be presented in the upcoming section.

## Assessment of hygiene competence

In order to be able to effectively assess hygiene competence in a standardized way in large samples [18], we use the principle of a *situational judgment test* [48], [49]. Such tests require a knowledge-based evaluation of realistically depicted scenes (e.g. pictures or short videos). Our test is focused on the ability to deliver a knowledge-based judgement of clinical situations regarding hygiene.

**Figure 1:**
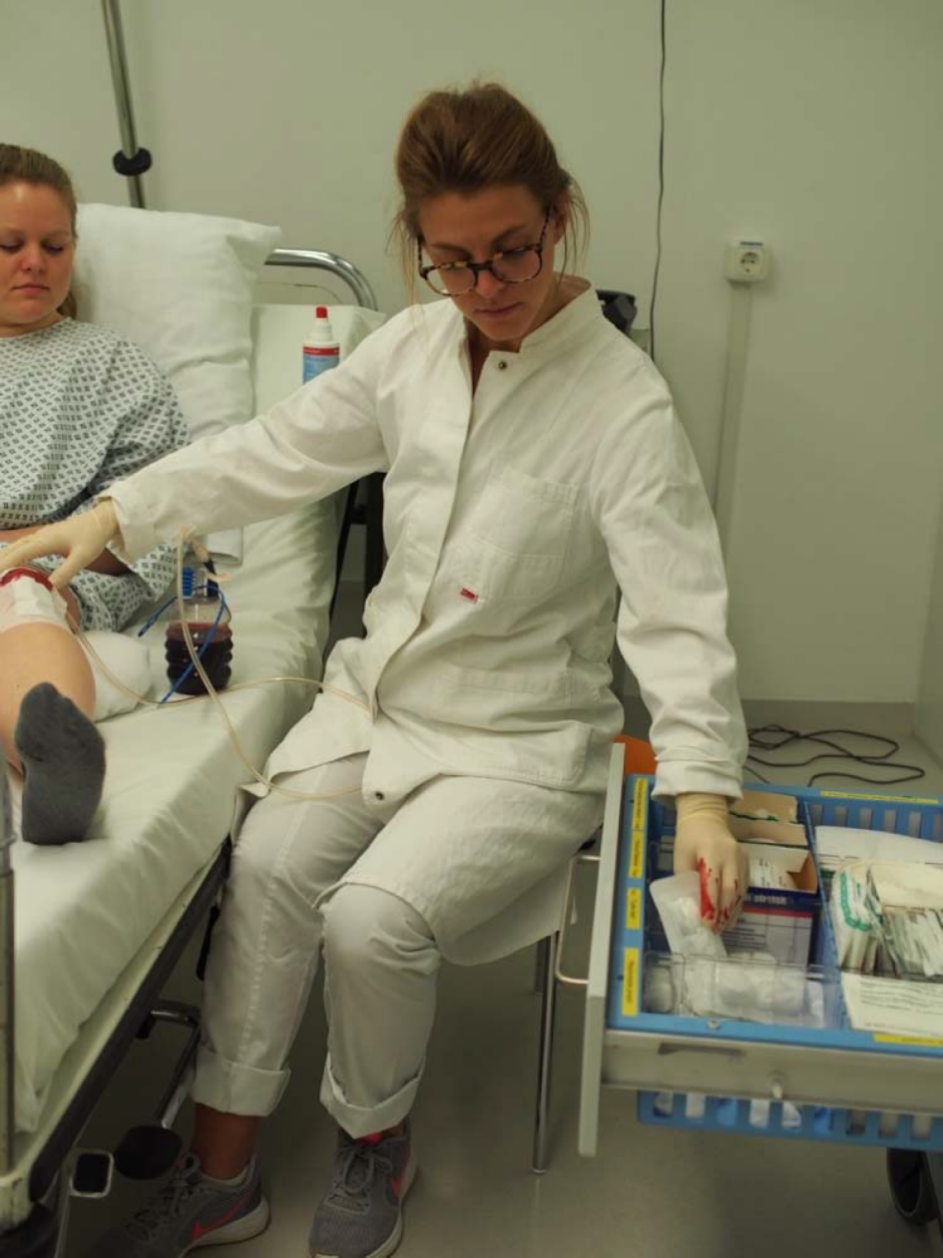
Sample picture vignette from the hygiene-SJT

In the sample vignette from the test (see figure 1 (Fig. 1)), a physician and a patient lying in a hospital bed can be seen. The physician is changing a bandage on the knee of the patient and meanwhile reaches into the drawer of a bandage supply chest with blood-contaminated gloves. This is a misconduct regarding hygiene rules, because the sterile bandage materials can be contaminated. During subsequent changings of bandages, germs can be transmitted to further patients via the contaminated material. Gloves must be taken off after possibly infectious activities and hands must be disinfected before touching further surfaces, objects, or persons.

On the basis of various picture vignettes, the test-takers are asked two questions for each vignette:

1. Do you perceive a hygiene-related problem in the clinical situation (Yes/No)?
2. Name and explain the hygiene-related problem (open text response).

For rating the answers given, both questions are taken into account. An item is scored as correct only if the right answer to question 1 (yes/no) is given along with a correct explanation.

By means of this test approach, it can be determined whether individuals are capable of applying (situation-unspecific) subject knowledge and knowledge about concrete clinical situations and behavioral processes to the picture vignettes in order to make correct judgements regarding the adherence to hygiene guidelines. Thus, two of the three aspects of the competence model described earlier are addressed in the test and are connected with each other: on the one hand, the aspect of knowledge, and on the other hand, the aspect of behavioral routines. Of course people who take the test do not perform any concrete clinical actions. Yet, the picture vignettes show snapshots of such actions, which are in turn evaluated by the test-takers. A question to be answered empirically is how well the test performance predicts the ability to adhere to hygiene standards in real clinical practice.

The third aspect of the described competence model – attitudes towards hygiene – is not focused by the SJT assessment. Available literature supports the conjecture that a discrepancy can exist between self-reported and observed hand hygiene [26], [32] and recommends observation in the context of clinical practice as the gold standard for assessing hygiene behavior [50]. When people working clinically rate their hand hygiene better than it is in reality, campaigns for the improvement of attitudes toward hygiene are not effective enough, since the target group already feels sufficiently qualified [26]. Whether the hygiene behavior of the target group has really improved through a training course will therefore be evident above all through observation of the behavior. Our test approach records whether relevant knowledge on hygiene is present that can be applied to situations in the clinical workday routine. This comes closer to a measurement of the results of hygiene processes than is possible through available tests of knowledge. Additionally, we propose to also assess attitudes towards hygiene through validated questionnaires. The use of established scales [12] is planned for this.

So far, the test approach described has been piloted in the form of an initial test version with 20 picture vignettes [51]; some initial results from this pilot study will be presented in the following. The aim of the study was to collect basic information on the usefulness of the test principle. The think aloud technique [52] was used as an established method of cognitive pretesting of instruments. Two students of medicine (age 21 and 23, both in the sixth semester) were confronted with the test material. They were asked to carry out the SJT and to verbalize all thoughts that went through their minds while working out the assignment. Generally, this procedure showed a good understanding of the test instructions and materials; the general test principle of identifying hygiene-related problems in the picture vignettes was plausible for the students. The thoughts verbalized by the students contained interesting hints regarding concrete questions that should be researched in further studies on the test materials. On the one hand, it was apparent that a lack of familiarity with certain medical work areas – e.g. the OR – went along with major difficulties to answer the items correctly. On the other hand, the test subjects could, in some cases, figure out correct answers through more prolonged, thorough contemplation of the situations during which they scrutinized the function of the activities and the protective measures depicted. This suggests that limiting the time available for judging the picture vignettes is a possibility to influence the difficulty of the test items. Furthermore, from these results, the question arises how context-specific hygiene competence is and to which degree hygienic work routines are transferable between different areas of medical activity. On the one hand, the idea of a simulation course on hygiene (see previous section) is based on teaching hygienic work routines by means of simulating concrete clinical situations. On the other hand, specific principles are thereby worked out, which should be transferable to other situations. Questions regarding didactic principles that enable the acquisition of transferable competences should be focused on in further studies. Furthermore, in the context of the pilot study, misconceptions in regards to hygienic work routines became apparent. In one picture vignette, a patient is shown, lying in her hospital bed and being pushed through a hallway by a care-giver. Her medical file is (hygienically correct) transported along in a plastic envelope, which is attached to the bedframe. A test-respondent explained that during her hospital internship, the medical files were mostly laid on the patient bed (which is hygienically incorrect). This indicates a lack of consciousness regarding the hygiene problem.

## Discussion

The present contribution argues in favor of considering hygiene as an essential component of clinical activity. If hygiene standards are not adhered to during clinical work, therapeutic goals are drawn into question because of a risk for the patients’ health. From the viewpoint of medical education research, this is the rationale for attempting to better understand which personal resources clinical staff use to work hygienically and to develop sound concepts for the promotion and the assessment of this competence. In order to reach this goal, the current contribution argues in favor of using modern, simulation based approaches of teaching as well as competence modeling and measurement.

The present empirical results based on the piloting of an initial version of the hygiene SJT show that the implemented test principle makes sense and is feasible. The content of the reflective processes initiated by means of the test are in line with the goal of the SJT, i.e., to provide a measure of the ability to assess clinical situations on the basis of knowledge about hygiene standards. Nonetheless, no reliable results can be reported on the basis of this first pilot study; further studies with larger samples are required. One limitation is that the SJT does not cover all aspects of hygiene competence. The assessment of concrete behaviors in clinical routine can only take place in practice settings and requires observation. Our initial results suggest, however, that hygiene perception and hygiene knowledge are sensible prerequisites for hygienically correct behavior in the workplace and that they can be assessed by means of the test.

Furthermore, the simulation-based concept for promotion of hygiene competence is only fully conclusive when it is embedded into a medical curriculum in which the other facets of competence are addressed, too – i.e. knowledge relevant for hygiene as well as corresponding attitudes. Future studies should therefore also investigate which relationships exist between the results of the SJT described here, existing hygiene knowledge tests, attitudes about hygiene and concrete performance in clinical situations.

## Note

^1^ The course was developed by PD Dr. Dirk Wilhelm (department of surgery at TUM MRI) and Prof. Dr. Rainer Burgkart (department of orthopedics and sport orthopedics at TUM MRI) and is part of the medical curriculum at the TUM since winter semester 2014/2015. 

## Acknowledgements

We would like to thank Michael Hanna, PhD, (mercury medical research & writing) for his assistance in translating the manuscript from German into English.

## Competing interests

The authors declare that they have no competing interests.

## Figures and Tables

**Table 1:**
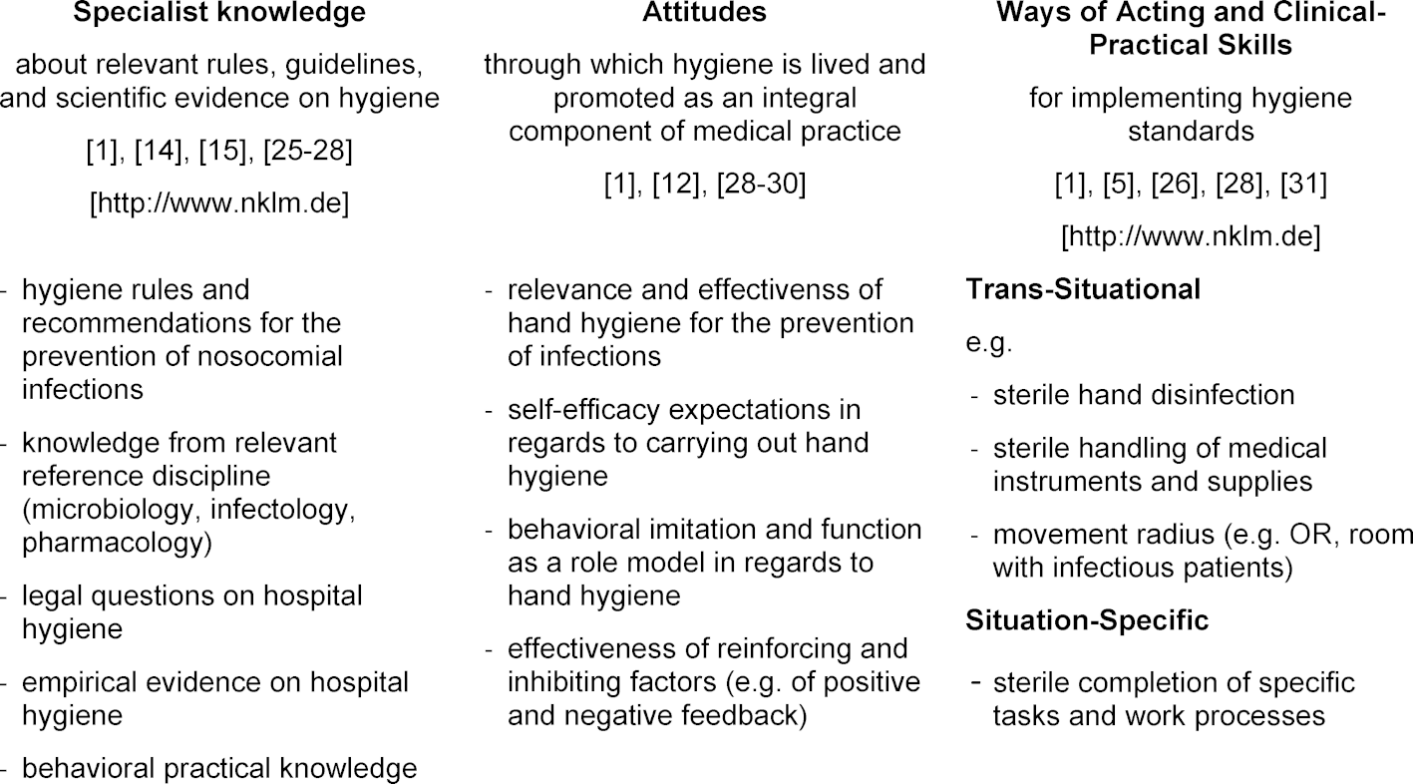
Hygiene competence as a multidimensional personal disposition

**Table 2:**
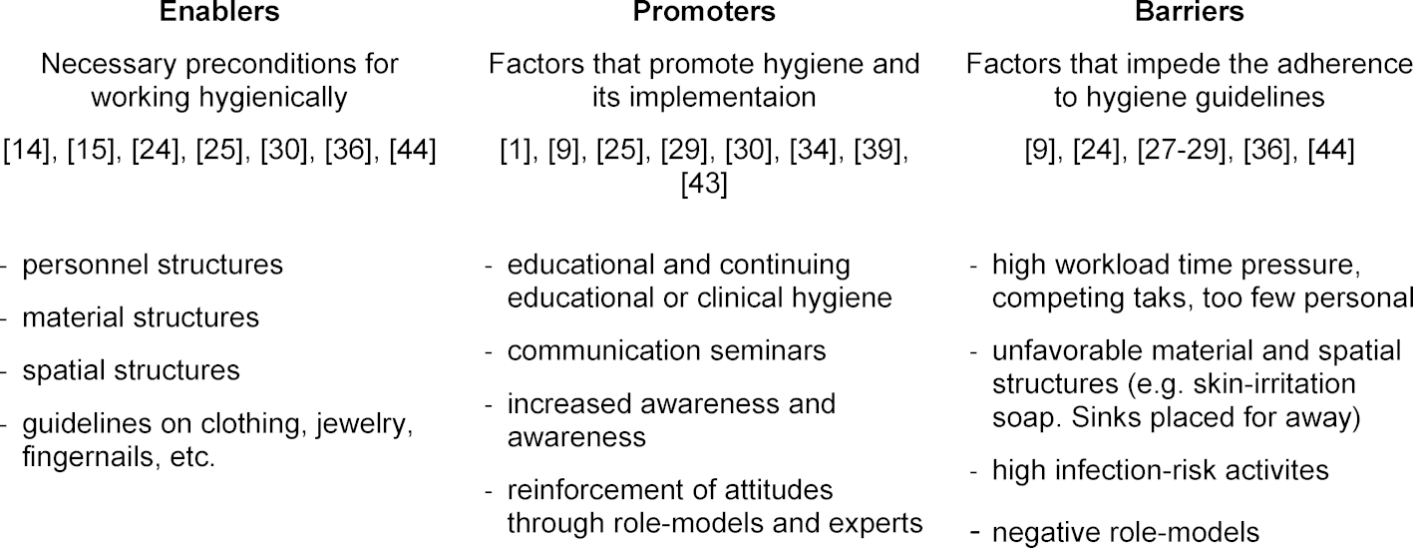
Factors that influence the implementation of hygienic ways of working in clinical contexts

**Table 3:**
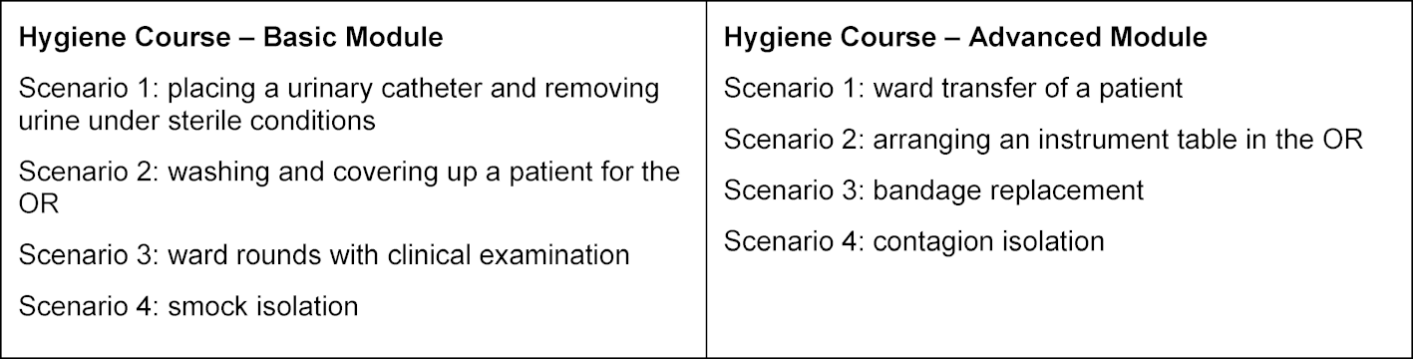
Overview of clinical situations simulated in the hygiene course, basic and advanced module
